# Complete Genome Sequences of Lytic Bacteriophages of *Xanthomonas arboricola* pv. Juglandis

**DOI:** 10.1128/genomeA.00336-16

**Published:** 2016-06-02

**Authors:** Julio Retamales, Ignacio Vasquez, Leonardo Santos, Cristopher Segovia, Manuel Ayala, Romina Alvarado, Pablo Nuñez, Javier Santander

**Affiliations:** a Agroadvance Laboratory, Peñaflor, Chile; b Microbial Pathogenesis and Vaccinology Research Group, Center for Genomics and Bioinformatics, Universidad Mayor, Santiago, Chile; c School of Biotechnology, Universidad Mayor, Santiago, Chile; d Ph.D. program in Integrative Genomics, Universidad Mayor, Santiago, Chile

## Abstract

Three bacteriophages, *f*20-Xaj*, f*29*-*Xaj, and *f*30-Xaj, with lytic activity against *Xanthomonas arboricola* pv. juglandis were isolated from walnut trees (VIII Bío Bío Region, Chile). These lytic bacteriophages have double-stranded DNA (dsDNA) genomes of 43,851 bp, 41,865 bp, and 44,262 bp, respectively. These are the first described bacteriophages with lytic activity against *X. arboricola* pv. juglandis that can be utilized as biocontrol agents.

## GENOME ANNOUNCEMENT

Current control of the Gram-negative bacteria *Xanthomonas arboricola* pv. juglandis, the causative agent of walnut blight disease, is mediated by the utilization of copper-based agrochemicals (1). Walnut blight is the main disease affecting walnut production (2), and losses during wet spring can exceed 80% if not controlled (3, 4).

Lytic bacteriophages with potential biocontrol activity against *X. arboricola* pv. juglandis have not been described. Here, we sequenced three lytic bacteriophages of *X. arboricola* pv. juglandis*.* DNA was purified using routine protocols (5). Sequencing was performed using the next-generation sequencer (NGS) Illumina MiSeq at Universidad Mayor, Center for Genomics and Bioinformatics (Huechuraba, Chile). The sequences were assembled using CLC Genomics Workbench 8.5.1 (Qiagen), resulting in a single contig with unknown ends. The genomic analysis was performed using the PHAST server (6). The assembled sequences were annotated by the National Center for Biotechnology Information (NCBI) Prokaryotic Genome Annotation Pipeline (PGAP; http://www.ncbi.nlm.nih.gov/genome/annotation_prok/). The summary report for the three sequenced bacteriophage genomes is shown in Table 1. Based on the predictions, these phage genomes contain genes for replication, structure, and lysis. Open reading frames (ORFs) were found for putative homing endonuclease, helicase, DNA ligase, and DNA polymerase. Bacteriophages *f*20-Xaj and *f*30-Xaj possess their own RNA polymerase, suggesting that they are T7 phage related. The ORFs for terminase, head morphogenesis protein, collar protein, putative tail tubular proteins, and tail fiber protein were found. Lysogenization genes, such as site-specific integrases and repressors, were not found. The ORFs for holin, lysozyme, and endolysin were also found. Alignment and molecular phylogenetic analysis by the maximum likelihood method (7, 8) showed that phages *f*20-Xaj and *f*30-Xaj are closely related to each other, having the same clade as *f*29-Xaj, *Xanthomonas campestris* pv. citri bacteriophages CP2 (GenBank accession no. NC_020205) and Cf1c (GenBank accession no. NC_001396.1) and *Xanthomonas* phage Prado (GenBank accession no. NC_022987).

**TABLE 1:** Summary report of the *de novo* assembly of the three Chilean *Xanthomonas arboricola* pv. juglandis bacteriophages from this study

Strain	BioSample no.	GenBank accession no.	G+C content (%)	Genome size (bp)	No. of CDSs^a^	Avg coverage (×)
f20-Xaj	SAMN04420465	KU595432	59.8	43,851	53	1,418
f29-Xaj	SAMN04420466	KU595434	49.8	41,865	61	370
f30-Xaj	SAMN04420467	KU595433	59.9	44,262	51	395

a CDSs, coding sequences.

### Nucleotide sequence accession numbers.

The genome sequences were deposited in DDBJ/EMBL/GenBank under BioProject PRJNA309087, and the accession numbers and details are listed in Table 1.
